# Right Atrial Epithelioid Angiosarcoma in a 44-Year-Old Woman: A Case Report

**DOI:** 10.7759/cureus.110238

**Published:** 2026-06-04

**Authors:** Jeet Shahani, Alexander Iribarne

**Affiliations:** 1 Biomedical Engineering, Binghamton University, Binghamton, USA; 2 Cardiac Surgery, Northwell Health, Staten Island, USA

**Keywords:** cardiac angiosarcoma, cardiac imaging-mri, cardiac surgery, epithelioid angiosarcoma, right atrial tumor

## Abstract

Primary cardiac angiosarcoma is a rare and aggressive malignancy that often presents with nonspecific symptoms, leading to delayed diagnosis. Early detection through multimodality imaging is essential for management.

A 44‑year‑old woman with no significant medical history presented with six weeks of vague symptoms that included intermittent retrosternal burning chest pain, palpitations, dyspnea, lightheadedness, and bilateral arm paresthesias. Outpatient evaluation revealed a suspicion for a right atrial mass on transthoracic echocardiogram. Her symptoms progressed, accompanied by episodes of bradycardia into the 40s, prompting emergency evaluation. Laboratory studies were largely unremarkable. CT angiography showed no pulmonary embolism, and staging CT scans revealed no metastases. Cardiac MRI demonstrated a 3.0 × 1.5 cm sessile right atrial mass with homogeneous enhancement. She underwent open-heart surgery with complete en bloc resection of the right atrial wall and reconstruction using bovine pericardium. Pathology confirmed epithelioid angiosarcoma. She had an uneventful postoperative recovery from her open-heart surgery. MRI brain and postoperative PET scan showed no evidence of residual disease or metastasis. She was offered adjuvant chemotherapy but chose close oncologic surveillance.

This case highlights the importance of multimodality imaging in diagnosing cardiac tumors, the feasibility of extensive right atrial resection with reconstruction, and the complexity of postoperative management in cardiac angiosarcoma.

## Introduction

Primary cardiac tumors are exceedingly rare, with an incidence of 0.02% in a large autopsy series [1]. More than 75% of these tumors are benign, and nearly half of the benign tumors are myxomas [1]. Metastatic tumors to the heart are more common than primary malignant cardiac tumors [1]. Among primary malignant cardiac tumors, angiosarcoma is the most common subtype, typically arising in the right atrium and occurring most frequently between the third and fifth decades of life, with a male‑to‑female ratio of approximately 2:1 [1]. The epithelioid variant is particularly aggressive, demonstrating early metastasis, and is difficult to detect and diagnose due to its epithelioid mesenchymal cell origin [2].

Cardiac angiosarcomas are highly aggressive, with metastases present in 47-89% of patients at diagnosis, most commonly to the lungs, but also to the brain, bone, liver, and colon [1,2]. Symptoms, particularly in early stages, are often nonspecific and include chest pain, dyspnea, malaise, fever, and arrhythmias or heart block, reflecting infiltration of the atrial wall or conduction system [3]. Because of this nonspecific presentation, diagnosis is frequently delayed, and prognosis remains poor.

Recent advancements that have shed light on the etiology and pathogenesis of cardiac angiosarcoma include intricate interaction among genetic, environmental, and cellular factors. Surgical resection along with adjuvant chemotherapy and radiotherapy has been the mainstay of treatment, with immunotherapy emerging as a promising avenue, with ongoing research focusing on the application of immune checkpoint inhibitors and combination therapies [3,4]. Targeted therapies tailored to address specific genetic alterations hold significant promise in improving patient outcomes and refining treatment strategies.

## Case presentation

A 44‑year‑old woman with no significant past medical history presented with six to seven weeks of vague intermittent burning retrosternal chest pain, palpitations, shortness of breath, lightheadedness, and bilateral arm paresthesia. Outpatient testing included a negative stress test and coronary calcium score. A transthoracic echocardiogram revealed a right atrial mass, prompting further evaluation. Her symptoms progressed, and she noted episodes of bradycardia into the 30s-40s, leading her to seek emergency care.

Emergency department evaluation

Laboratory studies as shown in Table 1 were notable for mild hyperkalemia, mildly elevated alanine transaminase (ALT), slightly elevated pro-B-type natriuretic peptide (proBNP), negative D‑dimer, and troponins that were not significantly elevated. Renal function was normal. Chest X‑ray showed no acute abnormalities.

**Table 1:** Initial laboratory values in emergency department workup. ALT: alanine transaminase; DDU: D-dimer units; BUN: blood urea nitrogen; proBNP: pro-B-type natriuretic peptide

Lab	Value	Reference Range
Potassium	5 mmol/L	3.5-5 mmol/L
ALT	54 U/L	0-41 U/L
proBNP	306 pg/mL	0-300 pg/mL
D-dimer	<150 ng/mL DDU	0-229 ng/mL DDU
Troponin	18 ng/L	0-13 ng/L
BUN	12 mg/dL	10-20 mg/dL
Serum creatinine	0.7 mg/dL	0.7-1.5 mg/dL

Imaging and diagnostic workup

CT angiogram chest (Figure 1) showed no pulmonary embolism, but showed an ill-defined, irregular filling defect in the infero-posterior aspect of the right atrium adjacent to the inferior vena cava (IVC), that appears to extend superiorly to the junction of the superior vena cava (SVC) and persisted on delayed imaging, compatible with an intracardiac mass measuring up to 4.5 cm from the SVC junction to the inferior aspect of the right atrium and 1.5 cm in diameter.

**Figure 1:**
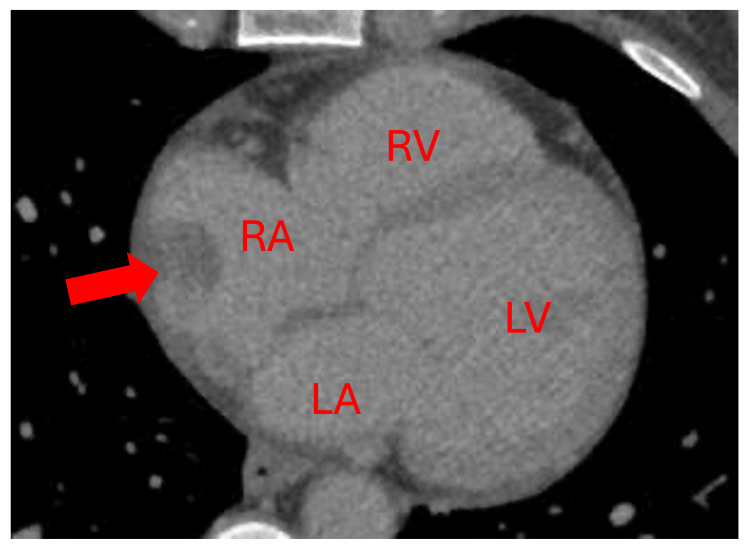
Axial CT of the heart showing the tumor extending into the right atrium. RA: right atrium; RV: right ventricle; LA: left atrium; LV: left ventricle; the arrow denotes the tumor.

CT of the neck, abdomen, and pelvis showed no metastatic disease. Cardiac catheterization was negative for coronary artery disease.

Cardiac MRI (Figure 2) demonstrated a well‑circumscribed, sessile right atrial mass measuring 3.0 × 1.5 cm, adherent to the inferoposterior right atrial wall and extending between the inferior and superior cavoatrial junctions without flow obstruction. The mass was predominantly T1 and T2 hypointense with diffuse internal enhancement on late gadolinium sequences. The homogeneous enhancement was inconsistent with thrombus. Biventricular systolic function was preserved (left ventricular ejection fraction (LVEF) of 75% and a right ventricular ejection fraction (RVEF) of 60%), with a small circumferential pericardial effusion noted.

**Figure 2:**
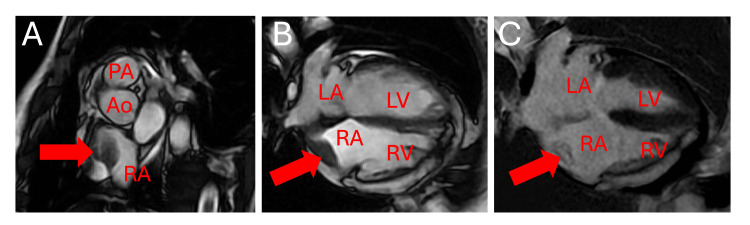
Cardiac MRI demonstrating a right atrial mass. (A) T2-weighted axial sequence; (B) T2-weighted rotated coronal sequence; (C) T1-weighted rotated coronal sequence. Ao: aorta; PA: pulmonary artery; RA: right atrium; RV: right ventricle; LA: left atrium; LV: left ventricle; the arrow denotes the tumor.

Surgical management

Open-heart surgery revealed a large, firm mass extending from the SVC to the IVC, completely adherent to the right atrial wall (Figure 3A). Intraoperative transesophageal echocardiogram confirmed tumor location (Figure 4A). En bloc resection of the entire right atrial free wall was required (Figure 3B), performed on cardiopulmonary bypass with bicaval venous cannulation and direct aortic cannulation; a beating-heart technique was selected to avoid unnecessary myocardial ischemic insult. Reconstruction was performed using a large bovine pericardial patch sewn to the remaining atrial rim with running 4‑0 Prolene sutures (Prolene®, Ethicon Inc., Raritan, NJ, USA) (Figure 3C). Intraoperative transesophageal echocardiogram confirmed complete resection of the tumor (Figure 4B). Total cardiopulmonary bypass time was 96 minutes. Estimated blood loss was 300 mL, requiring no transfusions.

**Figure 3:**
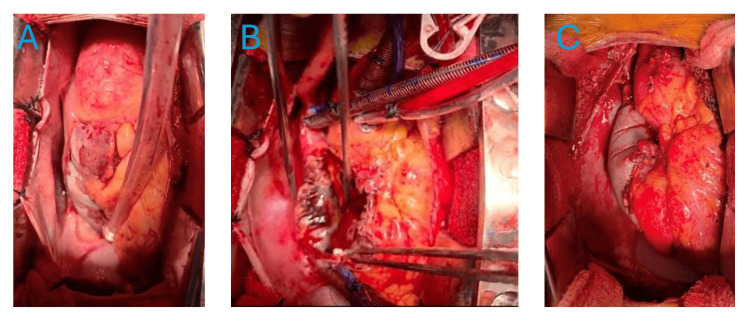
Intraoperative images. (A) Pre-excision image showing the desmoplastic reaction of the tumor on the right atrial wall. (B) Tumor clearly seen with the right atrial wall exposed longitudinally, on cardiopulmonary bypass. (C) Complete excision of the tumor and atrial reconstruction with a large bovine pericardial patch replacing the right atrial wall.

**Figure 4:**
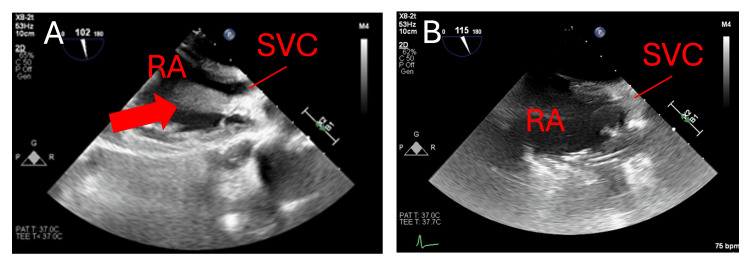
Transesophageal echocardiogram imaging obtained using bicaval view in M mode. (A) Pre-excision TEE highlighting the tumor extending into the right atrium. (B) Post-excision TEE confirming complete resection of the tumor. RA: right atrium; SVC: superior vena cava; TEE: transesophageal echocardiogram; the arrow denotes the tumor.

Postoperative course

The patient briefly developed junctional bradyarrhythmia requiring temporary pacing, likely attributable to surgical manipulation and reconstruction near the sinoatrial node, at the location of resection and patch repair. She had an otherwise uneventful post-open-heart-surgery recovery and was discharged on postoperative day 5. Outpatient brain MRI showed no suspicious lesions and no metastatic disease. One-month follow-up cardiac MRI showed no evidence of residual cardiac neoplasm. Right atrial morphology was suggestive of surgical reconstruction with mild diffuse thickening of the posterior right atrial wall with a mobility pattern suggestive of prosthetic material. There were no residual intracavitary masses, and both left ventricular (LV) and right ventricular (RV) function were preserved. PET scan demonstrated no residual or metastatic disease.

Pathology

Surgical pathology consisted of the largest specimen measuring 3.8 x 1.9 x 1.5 cm and remaining small fragments measuring 1.8 x 1.5 x 0.5 cm in aggregate (Figure 5).

**Figure 5:**
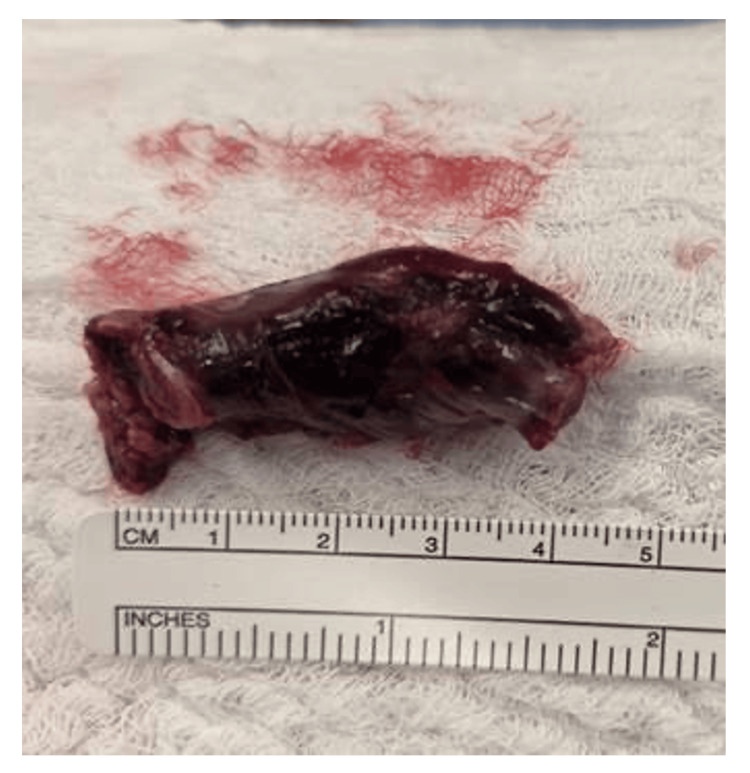
Pathologic specimen of the right atrial sarcoma measuring 3.9 cm in length.

Histopathology revealed a malignant vascular neoplasm consistent with epithelioid angiosarcoma, with 14 mitoses per 10 high‑power fields and 2% necrosis (Figures 6A, 6B). The extent of vaso-formation supported the diagnosis of epithelioid angiosarcoma, with a differential diagnosis being the so-called malignant epithelioid hemangioendothelioma.

**Figure 6:**
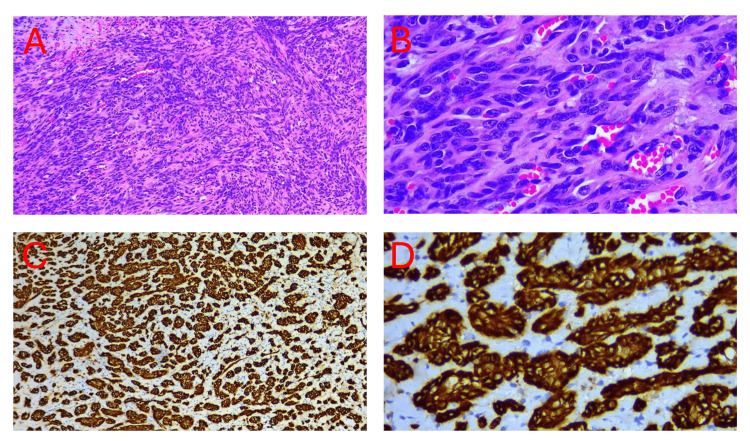
Pathology slides after excision of the tumor. (A) Low-magnification hematoxylin and eosin-stained tissue highlighting a vascular neoplasm. (B) High-magnification hematoxylin and eosin-stained tissue section highlighting malignant tumor cells. (C) Slide showing a CD31-positive stain. (D) Slide showing a CD34-positive stain.

Microscopic pathology showed a moderately cellular neoplasm involving cardiac muscle with focally myxoid stroma. Some areas are vasoformative, and others show small clusters and nests. The tumor cells are epithelioid with large nucleoli and irregular chromatin.

Performed immunostains show that the tumor cells are positive for ERG, CD31, and CD34 (Figures 6C, 6D) and negative for HHV-8, c-Myc, desmin, SMA, and ALK-D5F3. There is scattered weak expression of AE1/AE3.

Advanced genetic study using the Anchored Multiplex PCR (AMP^TM^; ArcherDX, Inc., Boulder, CO, USA) technology to detect gene fusions in tumor samples with the panel that consists of 129 cancer-related genes previously reported to be involved in chromosomal rearrangements was negative for gene fusions in the clinically validated panel. It was positive for the following gene fusions in the investigational panel: ATP9A::NFATC2, but the functional significance of this event is unknown.

Oncologic recommendations

The patient sought multiple opinions from various oncologists, and the case was presented at multiple tumor boards; the overall consensus was that the strain was resistant to chemotherapy and this portended an overall poor prognosis, while radiation treatment would be challenging due to the location in the heart. The patient was offered six cycles of AIM chemotherapy (doxorubicin (Adriamycin), ifosfamide, and mesna), a standard therapy used to treat soft tissue sarcomas, six to eight weeks postoperatively as a compromise solution. However, the patient chose to undergo close clinical and oncologic surveillance instead.

## Discussion

Primary cardiac angiosarcoma is known to be rare and highly aggressive. Cardiac tumors often produce no symptoms but may interfere with blood flow or valvular function, and arrhythmias such as bradycardia may arise due to myocardial infiltration [5]. These tumors preferentially arise in the right atrium, typically in patients in their 30s to 50s, and show a male predominance [1]. Metastatic disease is present in 47-89% of cases at diagnosis [1,2,4-6]. Common metastatic sites include the lungs (20-55%), liver (10-22%), and bone (10-20%) [3-7]. Several case reports describe rapid progression and early mortality, including patients who died within months despite intervention [8].

Conversely, rare cases of sustained remission following complete resection have been reported, particularly in patients without metastases [9,10]. Prognosis remains poor. A multinational retrospective review reported a median overall survival (OS) of 17.5 months, with improved outcomes in patients undergoing R0 resection (34.8 months) compared with R1/R2 resection (18.3 months) or no surgery (8.9 months) [4]. Survival has not improved in contemporary cohorts [4]. Systematic reviews report mortality rates of 65-100% and median survival of 5-26 months, with worse outcomes in metastatic disease [1-5,7].

This case is notable for early detection despite nonspecific symptoms, absence of metastatic disease on CT, MRI brain, and PET, and complete en bloc resection of the right atrial wall. Pathology confirmed epithelioid angiosarcoma, a rare variant. Although adjuvant chemotherapy is commonly recommended, evidence is limited and largely extrapolated from soft‑tissue sarcoma management [11]. Contemporary reviews emphasize multimodality treatment, including surgery, systemic therapy, and surveillance imaging [12], with complete resection being the most effective predictor of successful outcomes. This case adds to the limited literature describing right atrial epithelioid angiosarcoma in a female patient with early detection, complete resection, and no metastatic disease on comprehensive imaging. Notably, the male-to-female predominance in this primary cardiac angiosarcoma further distinguishes this case, as female presentations remain underrepresented in the literature, and their clinical behavior is not yet well characterized.

## Conclusions

Our patient presented with vague cardiac symptoms that were promptly evaluated, leading to an early diagnosis. A combination of echocardiography and advanced imaging, including cardiac MRI, was essential in establishing the diagnosis. Excisional biopsy with histopathological analysis and immunohistochemical staining subsequently confirmed the diagnosis of primary cardiac angiosarcoma. Fortunately, the patient underwent complete surgical resection, which offered her the greatest chance of survival in the setting of this highly malignant primary cardiac angiosarcoma. This case highlights the importance of early multimodality imaging, aggressive surgical management, and individualized oncologic planning in the treatment of this rare and aggressive malignancy.
